# Body Mass Index at Diagnosis, Prediagnostic Weight Loss, and Survival in Colorectal Cancer

**DOI:** 10.1001/jamanetworkopen.2026.29571

**Published:** 2026-08-14

**Authors:** Marko Mandic, Durgesh Wankhede, Fatemeh Safizadeh, Michael Hoffmeister, Hermann Brenner

**Affiliations:** 1 Cancer Prevention Graduate School, German Cancer Research Center (DKFZ), Heidelberg, Germany; 2 Medical Faculty Heidelberg, Heidelberg University, Heidelberg, Germany; 3 Division of Clinical Epidemiology of Early Cancer Detection, German Cancer Research Center (DKFZ), Heidelberg, Germany

## Abstract

**Importance:**

Evidence on the association of excess adiposity with colorectal cancer (CRC) prognosis is conflicting, with some studies reporting better survival in patients with elevated body mass index (BMI)—often referred to as the obesity paradox.

**Objective:**

To investigate to what extent the obesity paradox can be explained by prediagnostic cancer-associated weight loss.

**Design, Setting, and Participants:**

This multicenter, population-based case-control study with prospective follow-up included patients aged 30 years or older with histologically confirmed CRC who were recruited from more than 20 hospitals in southwest Germany between 2001 and 2016, with survival follow-up through September 2018. Analysis was conducted from September 2025 to June 2026.

**Exposure:**

BMI at diagnosis (underweight, BMI <18.5; normal weight, BMI 18.5 to <25.0; overweight, BMI 25.0 to <30.0; obesity, BMI ≥30.0), and percent weight change during the 5 to 14 years preceding diagnosis, both derived from self-reported height and body weight.

**Main Outcomes and Measures:**

Overall, CRC-specific, and recurrence-free survival were assessed using Cox proportional hazards regression models adjusted for demographic and lifestyle factors, tumor characteristics, and comorbidities, estimated as hazard ratios (HRs) with 95% CIs.

**Results:**

Among 5521 patients with CRC (2159 female [39%]; median [IQR] age at diagnosis, 69 [61-76] years), 2429 deaths occurred during a median (IQR) follow-up of 5.2 (3.4-9.9) years, including 1381 CRC-related deaths. Compared with patients with normal weight at diagnosis, those who had overweight had modestly higher overall survival (HR, 0.89; 95% CI, 0.81-0.98), while no association was observed for obesity. Prediagnostic weight loss of more than 10% was associated with worse survival (HR, 1.55; 95% CI, 1.39-1.72). Upon mutual adjustment for at-diagnosis BMI and prediagnostic weight change, associations for overweight and obesity were attenuated to null.

**Conclusions and Relevance:**

In this case-control study with prospective follow-up of patients with CRC, the obesity paradox appeared to be largely attributable to prediagnostic cancer-associated weight loss. The apparent survival advantage associated with overweight and obesity at diagnosis should not be interpreted as evidence to encourage weight gain for patients with healthy weight or to revise existing survivorship recommendations.

## Key Points

**Question:**

To what extent is the obesity paradox in colorectal cancer (CRC)—the survival advantage associated with elevated body mass index—explained by prediagnostic cancer-associated weight loss?

**Findings:**

In this case-control study of 5521 patients with CRC, the survival benefit associated with overweight and obesity at diagnosis was largely attenuated after accounting for prediagnostic weight loss. Conversely, weight loss of more than 10% before diagnosis was associated with worse survival outcomes.

**Meaning:**

These findings suggest that the apparent survival advantage associated with overweight and obesity at diagnosis may be explained by prediagnostic weight loss.

## Teaser

This case-control study examines the association of prediagnostic weight loss with survival among patients with colorectal cancer.

## Introduction

Colorectal cancer (CRC) is the third most commonly diagnosed malignant tumor and the second leading cause of cancer-related mortality worldwide, with 1.9 million new cases and 900 000 deaths in 2022.^1^ Advances in CRC screening and treatment, combined with aging populations have already led to a substantial growth in the number of CRC survivors—a trend projected to continue in the coming decades.^2^ Overweight and obesity, typically defined as body mass index (BMI) thresholds of 25 to <30 and ≥30^2,3^ respectively, are well-established risk factors for CRC.^4^ In 2022, an estimated 2.5 billion adults had overweight, including approximately 890 million living with obesity.^5^

However, the association of excess weight with CRC prognosis has been the subject of much debate in the past decade. Several studies have reported better survival among patients with higher body weight, a phenomenon often referred to as the obesity paradox.^6^ Proposed explanations include less aggressive tumor biology in individuals with obesity, detection bias (ie, people with excess weight go to medical appointments more often), and the protective effect of fatty tissue as a nutrient reserve during cancer treatment.^7^ On the other hand, it has also been argued that much of the paradox can be attributed to methodological limitations of previous epidemiological studies, including reverse causation, collider bias, and inadequate adjustment for confounding factors.^8^ Notably, unintentional weight loss in the months and years preceding CRC diagnosis is common^9,10,11,12^ and has been associated with poorer survival outcomes.^13^ Despite this, prior research examining the association of excess weight with CRC survival has not consistently accounted for cancer-related weight loss, which may influence the observed association. In this study, using a large cohort of patients with CRC with long-term follow-up, we aimed to investigate the extent to which the obesity paradox in CRC can be explained by prediagnostic weight loss bias.

## Methods

This case-control study was approved by the ethics committees of Heidelberg University and the state medical boards of Baden-Württemberg and Rhineland-Palatinate. The study followed the Strengthening the Reporting of Observational Studies in Epidemiology (STROBE)^14^ reporting guideline.

### Study Design and Population

**Figure 1.**
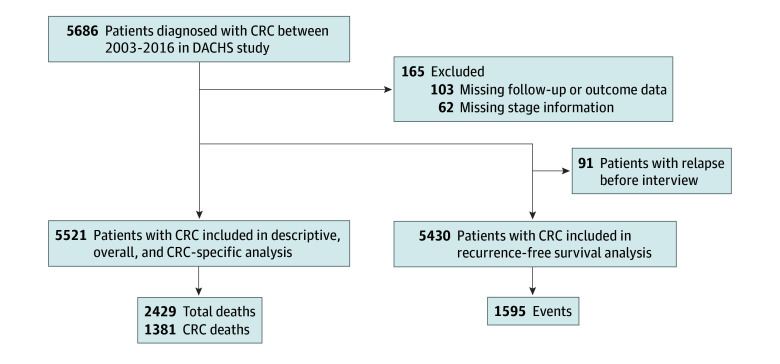
Flow Diagram of Selection of the Study Population CRC indicates colorectal cancer; DACHS, Darmkrebs: Chancen der Verhütung Durch Screening (Colorectal Cancer: Opportunities for Prevention Through Screening).

Our prospective patient-cohort analysis uses data from the DACHS study (The Darmkrebs: Chancen der Verhütung Durch Screening [Colorectal Cancer: Chances of Prevention Through Screening]), a population-based case-control study with follow-up of cases.^15,16^ Patients aged 30 years or older with histologically confirmed CRC (*International Statistical Classification of Diseases and Related Health Problems, Tenth Revision [ICD-10]* codes C18-C20) were recruited between 2001 and 2016 in more than 20 clinics in southwest Germany. Patients were informed about the study by their attending physicians, usually during or shortly after their hospital stay for CRC surgery. Approximately 50% of all eligible cases in the study area were recruited. Before enrollment, written informed consent was obtained from all participants. The flowchart of the selection of the study population is displayed in Figure 1. Of the 5686 patients with CRC recruited to the study, 165 were excluded due to missing outcome or stage information. For the recurrence-free survival (RFS) analysis, an additional 91 participants with relapse before baseline interview were excluded.

### Data Collection

Trained interviewers collected sociodemographic, medical, and lifestyle data via a standardized questionnaire (median [IQR] time from diagnosis to interview, 21 [9-182] days). Inpatient treatment information was collected from the hospitals. Cases were followed up at 3, 5, and 10 years after recruitment using standardized questionnaires. Deaths were identified during the follow-up, and causes of death were identified from death certificates collected from the local public health departments. Follow-up data have been described previously.^17^

### Exposure Ascertainment

Participants reported their weight (kilograms) and height (centimeters) at diagnosis, and past weight at each decennial age starting age 20 years. BMI at diagnosis (calculated as weight in kilograms divided by height in meters squared) was categorized as underweight (BMI <18.5), normal weight (BMI 18.5 to <25.0), overweight (BMI 25.0 to <30), obesity class I (BMI 30.0 to <35.0), and obesity class II and III (BMI of 35.0 or greater).^3^ To investigate how disease-related weight loss may have affected the association of BMI with survival, we calculated weight change since at least 5 years before diagnosis. We chose this timeframe because CRC has been shown to spend, on average, 2 to 6 years in a preclinical phase before diagnosis.^18,19,20^ Because previous weight was recalled for decennial ages (eTable 1 in Supplement 1), weight change referred to different intervals before diagnosis (5 to 14 years before diagnosis). Weight change was categorized as no weight loss or weight gain, 0.1% to 5.0% weight loss, 5.1% to 10.0% weight loss, and greater than 10.0% weight loss.

### Statistical Analysis

Patient characteristics were presented using descriptive statistics for the total cohort and by BMI category. Cox proportional hazards models were used to evaluate the association of BMI at diagnosis and weight change since 5 to 14 years before diagnosis with overall survival (OS), CRC-specific survival (CSS), and RFS. Days since diagnosis was used as the underlying time scale. Because patients entered the study after their cancer diagnosis, delayed-entry (left-truncated) models were fitted. The end point for OS was death from any cause, the end point for CSS was death from CRC, and the end point for RFS was CRC recurrence or death from CRC. For CSS and RFS, cause-specific Cox models were used, with deaths from causes other than CRC treated as competing events and individuals censored at the time of the competing event. Follow-up ended on September 9, 2018. Two adjustment levels were used in the Cox models: (1) adjustment for age and sex and (2) additional adjustment (main model) for potential confounders including cancer stage, tumor location, tumor grade, physical activity, smoking status, nonsteroidal anti-inflammatory drug (NSAID) use, statin use, hyperlipidemia, history of cardiovascular disease (CVD), diabetes, history of other cancer, and use of chemotherapy and radiotherapy. Time-dependent interaction terms addressed proportional hazards deviations.

We evaluated the association of BMI at diagnosis and weight change since 5 to 14 years before diagnosis with survival. To explore their interplay, we also employed models with mutual adjustment. Potential nonlinear dose-response associations were explored using restricted cubic spline models.

We performed subgroup analyses stratified by sex, cancer stage, and smoking status. Due to fewer number of exposed cases, obesity categories were merged (BMI ≥30). Sensitivity analyses included restricting the analysis to patients with weight information 5 to 7 years before diagnosis, excluding the first year of follow-up (landmark analysis), and excluding patients with stage IV cancer. Finally, to directly capture the effect of prediagnostic BMI downward shift due to weight loss, we assessed survival according to BMI trajectory from 5 to 14 years before diagnosis, categorized as normal BMI to normal BMI, normal BMI to underweight BMI, overweight BMI to overweight BMI, and overweight BMI to normal BMI.

Missing data were handled by multiple imputation using R mice package (eMethods in Supplement 1).^21^ All statistical tests were 2-sided, and a P value less than .05 was considered to indicate statistical significance. Analysis was conducted September 2025 to June 2026. All analyses were conducted using R software version 4.5.1 (R Project for Statistical Computing).

## Results

The final cohort included 5521 patients (2159 female [39%]; median [IQR] age, 69 [61-76] years). Characteristics of the participants stratified according to BMI at diagnosis are presented in Table 1. The median (IQR) BMI at the time of diagnosis was 26.1 (23.6-29.0). The distribution of BMI at diagnosis and weight change since 5 to 14 years before diagnosis is shown in eFigure 1 in Supplement 1. Weight change over the 5 to 14 years preceding diagnosis showed a slight shift toward weight loss with shorter time intervals (eFigure 2 in Supplement 1), and weight loss increased from stage I to stage IV disease (eFigure 3 in Supplement 1).

**Table 1.** Characteristics of the Study Population Overall and by BMI at Diagnosis

Variable	Participants, No. (%)					
	Total (N = 5521)	BMI at diagnosis^a^				
		<18.5 (n = 114)	18.5 to <25.0 (n = 1970)	25.0 to <30.0 (n = 2323)	30.0 to <35.0 (n = 855)	≥35.0 (n =230)
Sex						
Female	2159 (39)	88 (77)	902 (46)	766 (33)	312 (36)	112 (49)
Male	3362 (61)	26 (23)	1068 (54)	1557 (67)	543 (64)	118 (51)
Age at diagnosis, median (IQR), y	69 (61 to 76)	70 (60 to 81)	69 (60 to 77)	70 (62 to 77)	69 (61 to 75)	66 (60 to 73)
Weight change since 5-14 y before diagnosis, median (IQR), %^b^	−1.4 (−7.6 to 2.7)	−12.8 (−21.4 to −7.5)	−4.8 (−10.3 to 0.0)	0.0 (−5.9 to 2.9)	2.0 (−3.5 to 7.8)	4.5 (−1.0 to 14.6)
Tumor location						
Colon	3304 (60)	66 (58)	1156 (59)	1370 (59)	544 (64)	153 (66)
Rectum	2217 (40)	48 (42)	814 (41)	953 (41)	311 (36)	77 (33)
TNM stage						
I	1266 (23)	16 (14)	411 (21)	575 (25)	208 (24)	52 (23)
II	1665 (30)	31 (27)	584 (30)	708 (30)	260 (30)	71 (31)
III	1752 (32)	45 (33)	655 (33)	708 (30)	264 (31)	74 (32)
IV	838 (15)	22 (16)	320 (16)	332 (14)	123 (14)	33 (14)
Tumor grade^c^						
I	140 (3)	2 (2)	45 (2)	71 (3)	12 (1)	7 (3)
II	3840 (71)	88 (78)	1341 (69)	1621 (71)	604 (72)	165 (72)
III	1111 (20)	14 (12)	430 (22)	448 (20)	171 (20)	44 (19)
IV	20 (0)	1 (1)	4 (0)	7 (0)	6 (0)	2 (1)
Unknown	327 (6)	8 (7)	123 (6)	139 (5)	46 (5)	11 (5)
Physical activity, median (IQR), MET-h/wk^d^	183 (125 to 256)	182 (124 to 251)	176 (119 to 240)	185 (126 to 261)	198 (140 to 284)	177 (124 to 252)
Smoking status^e^						
Never smoker	2446 (44)	55 (49)	872 (45	1027 (44)	374 (44)	100 (43)
Former smoker	2200 (40)	23 (20)	714 (36)	978 (42)	377 (44)	102 (44)
Current smoker	845 (15)	35 (31)	371 (19)	305 (13)	102 (12)	29 (13)
Statin use^f^	889 (16)	4 (3)	255 (13)	401 (17)	175 (20)	50 (22)
Nonsteroidal anti-inflammatory drug use^g^	1437 (26)	16 (14)	406 (21)	646 (28)	260 (31)	92 (40)
Hyperlipidemia^h^	1598 (30)	20 (19)	495 (26)	706 (32)	294 (36)	75 (34)
Diabetes^i^	1016 (18)	8 (7)	239 (12)	446 (19)	229 (27)	85 (37)
History of cardiovascular disease^j^	1311 (24)	21 (18)	413 (21)	562 (24)	239 (28)	70 (30)
History of other cancer^k^	692 (13)	12 (10)	248 (13)	295 (13)	113 (13)	23 (10)
Chemotherapy^l^	2473 (45)	51 (45)	934 (47)	1016 (44	367 (42)	100 (44)
Radiotherapy^m^	961 (17)	17 (15)	375 (19)	401 (17)	133 (16)	33 (14)

Abbreviations: BMI, body mass index; MET, metabolic equivalent of task.

^a^ BMI was calculated as weight in kilograms divided by height in meters squared; there were 29 participants who had a missing value for BMI at diagnosis.

^b^ Missing value for 144 participants.

^c^ Missing value for 83 participants.

^d^ Missing value for 76 participants.

^e^ Missing value for 30 participants.

^f^ Missing value for 5 participants.

^g^ Missing value for 68 participants.

^h^ Missing value for 245 participants.

^i^ Missing value for 42 participants.

^j^ Defined as a self-reported history of heart failure, myocardial infarction, angina pectoris, or stroke.

^k^ Missing value for 20 participants.

^l^ Chemotherapy and radiotherapy administered before relapse or metastasis, respectively; there were 7 missing values for both variables.

^m^ Chemotherapy and radiotherapy administered before a relapse or metastasis, respectively; there were 7 missing values for both variables.

During a median (IQR) follow-up of 5.2 (3.4-9.9) years, there were a total of 2429 deaths and 1381 CRC-related deaths. In the RFS analysis, there were a total of 1595 events during a median (IQR) follow-up of 4.5 (2.3-6.5) years.

### Association of BMI at Diagnosis and Prediagnostic Weight Change With Survival

Associations of BMI at diagnosis and weight change since 5 to 14 years before diagnosis with survival are shown in Table 2. Compared with patients with normal weight, patients who had overweight at diagnosis had a better OS (hazard ratio [HR], 0.89; 95% CI, 0.81-0.98). Similar inverse associations were observed among patients with obesity, although the estimates did not reach statistical significance (class I obesity: HR, 0.90; 95% CI, 0.79-1.02; class II-III obesity: HR, 0.89; 95% CI, 0.71-1.11). Patients with underweight at diagnosis had worse OS (HR, 1.40; 95% CI, 1.09-1.80). Comparable estimates were observed for CSS and RFS, although the lower number of events limited statistical power. Relative to patients who experienced no weight loss or gained weight, those who lost more than 10% of their body weight had worse OS (HR, 1.55; 95% CI, 1.39-1.72), CSS (HR, 1.51; 95% CI, 1.31-1.74), and RFS (HR, 1.33; 95% CI, 1.17-1.52).

**Table 2.** Association of BMI at Diagnosis and Prediagnostic Weight Change With Survival

Survival outcome	Patients, No.	Events, No.	HR (95% CI)		
			Age- and sex-adjusted^a^	Multivariable^b^	Mutually adjusted, multivariable^c^
Overall survival					
BMI at diagnosis^d^					
<18.5	114	65	1.51 (1.17-1.94)	1.40 (1.09-1.80)	1.22 (0.93-1.60)
18.5 to <25.0	1986	895	1 [Reference]	1 [Reference]	1 [Reference]
25.0 to <30.0	2332	1013	0.86 (0.79-0.95)	0.89 (0.81-0.98)	0.95 (0.87-1.05)
30.0 to <35.0	858	368	0.92 (0.82-1.04)	0.90 (0.79-1.02)	1.01 (0.89-1.15)
≥35.0	231	88	0.90 (0.73-1.13)	0.89 (0.71-1.11)	1.06 (0.84-1.33)
Weight change since 5-14 y before diagnosis, %					
No weight loss	2552	957	1 [Reference]	1 [Reference]	1 [Reference]
0.1%-5.0%	1008	364	1.03 (0.91-1.15)	0.99 (0.88-1.12)	0.98 (0.87-1.10)
5.1%-10.0%	988	392	1.18 (1.05-1.32)	1.04 (0.93-1.17)	1.03 (0.92-1.16)
>10.0%	973	612	1.89 (1.70-2.10)	1.55 (1.39-1.72)	1.54 (1.37-1.72)
CRC-specific survival					
BMI at diagnosis^d^					
<18.5	114	38	1.52 (1.09-2.11)	1.48 (1.06-2.06)	1.32 (0.83-1.86)
18.5 to <25.0	1986	512	1 [Reference]	1 [Reference]	1 [Reference]
25.0 to <30.0	2332	576	0.90 (0.79-1.01)	0.94 (0.83-1.06)	1.00 (0.88-1.13)
30.0 to <35.0	858	202	0.89 (0.75-1.04)	0.92 (0.78-1.09)	1.04 (0.86-1.24)
≥35.0	231	53	0.88 (0.66-1.17)	1.00 (0.75-1.33)	1.19 (0.88-1.60)
Weight change since 5-14 y before diagnosis, %					
No weight loss	2552	564	1 [Reference]	1 [Reference]	1 [Reference]
0.1%-5.0%	1008	205	0.96 (0.81-1.12)	0.85 (0.72-1.00)	0.85 (0.72-1.00)
5.1%-10.0%	988	267	1.29 (1.11-1.49)	0.99 (0.85-1.15)	0.98 (0.84-1.14)
>10.0%	973	345	2.00 (1.74-2.29)	1.51 (1.31-1.74)	1.49 (1.29-1.73)
Recurrence-free survival					
BMI at diagnosis^d^					
<18.5	114	47	1.54 (1.13-2.10)	1.45 (1.07-1.98)	1.31 (0.95-1.82)
18.5.0 to <25.0	1986	618	1 [Reference]	1 [Reference]	1 [Reference]
25.0 to <30.0	2332	697	0.92 (0.82-1.03)	0.97 (0.87-1.09)	1.02 (0.91-1.15)
30.0 to <35.0	858	259	0.97 (0.83-1.12)	1.04 (0.89-1.21)	1.13 (0.96-1.33)
≥35.0	231	64	0.89 (0.68-1.16)	0.99 (0.76-1.29)	1.12 (0.85-1.48)
Weight change since 5-14 y before diagnosis, %					
No weight loss	2552	711	1 [Reference]	1 [Reference]	1 [Reference]
0.1%-5.0%	1008	267	1.00 (0.86-1.16)	0.91 (0.78-1.06)	0.91 (0.79-1.06)
5.1%-10.0%	988	316	1.20 (1.04-1.38)	0.95 (0.82-1.09)	0.95 (0.82-1.10)
>10.0%	973	391	1.72 (1.51-1.96)	1.33 (1.17-1.52)	1.34 (1.16-1.54)

Abbreviations: BMI, body mass index; CRC, colorectal cancer; HR, hazard ratio.

^a^ Adjusted for age (continuous) and sex. Additional adjustment for age × log(time) to account for time-dependent effects.

^b^ Additionally adjusted for cancer stage (I to IV), tumor location (colon, rectum), tumor grade (G1, G2, G3, G4, or unknown), physical activity (continuous; average lifetime metabolic equivalent of task hours per week), smoking status (never, previous, or current), nonsteroidal anti-inflammatory drug (NSAID) use (yes or no; defined as current regular use of NSAIDs, including aspirin, ≥2 times per week), statin use (yes or no; defined as current use at least 1 time per week), hyperlipidemia (yes or no), history of cardiovascular disease (heart failure, myocardial infarction, angina pectoris, or stroke; yes or no), diabetes (yes or no), history of other cancer (yes or no), and use of chemotherapy and radiotherapy (yes or no; administered before a relapse or metastasis). Time interval (years before diagnosis) included in models that include weight change since 5 to 14 years before diagnosis.

^c^ Fully adjusted and simultaneous adjustment for BMI at diagnosis and weight change since 5 to 14 years before diagnosis.

^d^ Calculated as weight in kilograms divided by height in meters squared.

In models that simultaneously included BMI at diagnosis and prediagnostic weight change, no significant associations were found for overweight (HR, 0.95; 95% CI, 0.87-1.05) and obesity (class I obesity: HR, 1.01; 95% CI, 0.89-1.15; class II-III obesity: HR, 1.06; 95% CI, 0.84-1.33), while the association for weight loss of greater than 10% remained (HR, 1.54; 95% CI, 1.37-1.72]).

Dose-response associations of BMI at diagnosis and weight change since 5 to 14 years before diagnosis with OS, CSS, and RFS, explored using restricted cubic splines models, are shown in eFigure 4 in Supplement 1. Relative to a BMI of 25.0, BMI values in the normal weight and underweight range (<25.0) were associated with worse survival outcomes, whereas BMI values in the overweight and obesity range were associated with lower or no difference in risk. The spline analyses suggested that the associations with the largest magnitude were observed for increasing levels of prediagnostic weight loss.

### Subgroup and Sensitivity Analyses

**Figure 2.**
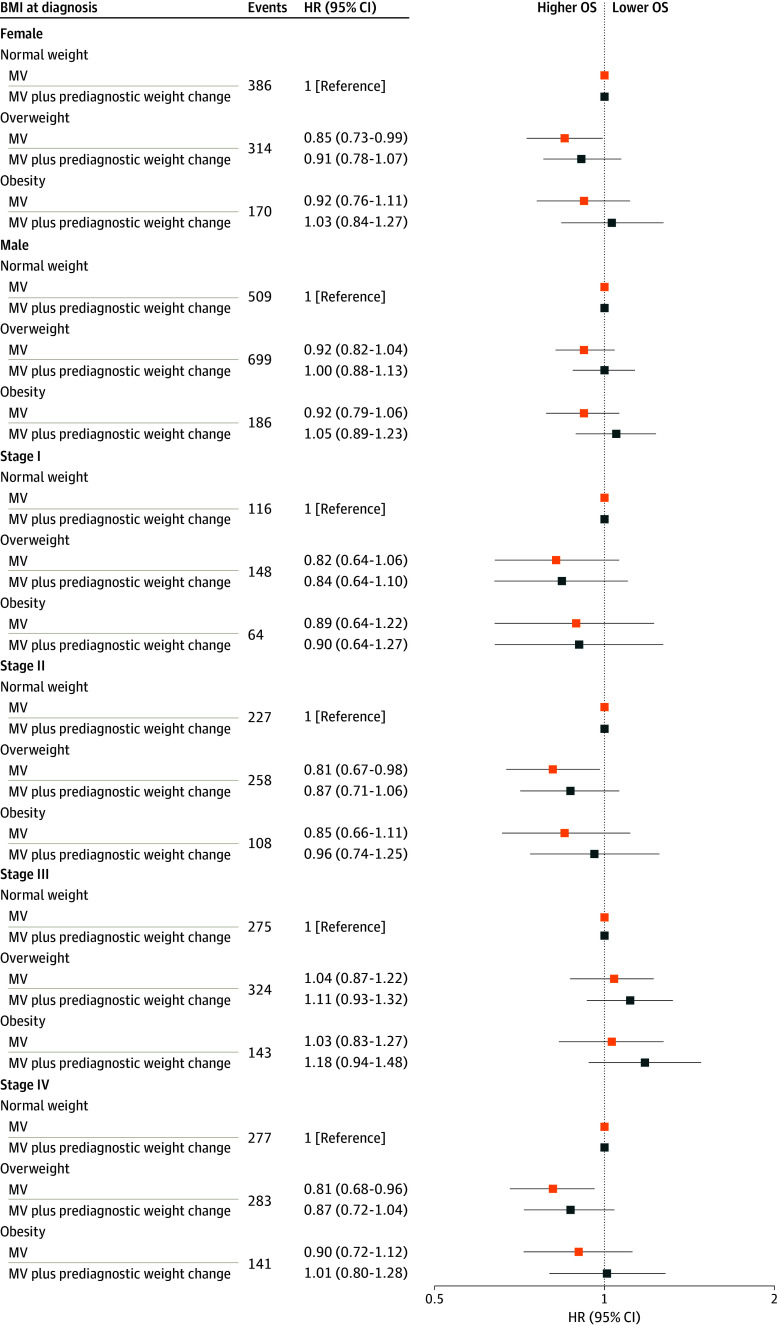
Descriptive Forest Plot of Body Mass Index (BMI) at Diagnosis and Overall Survival (OS), Stratified by Sex and Stage The multivariable (MV) models adjusted for age, sex, tumor stage, tumor grade, tumor location, physical activity, smoking, nonsteroidal anti-inflammatory drugs and statin use, hyperlipidemia, history of cardiovascular disease (heart failure, myocardial infarction, angina pectoris, or stroke), diabetes, history of other cancer, and chemotherapy and radiotherapy. The MV plus prediagnostic weight change model additionally adjusted for weight change since 5 to 14 years before diagnosis and the corresponding time interval (years before diagnosis). HR indicates hazard ratio.

**Figure 3.**
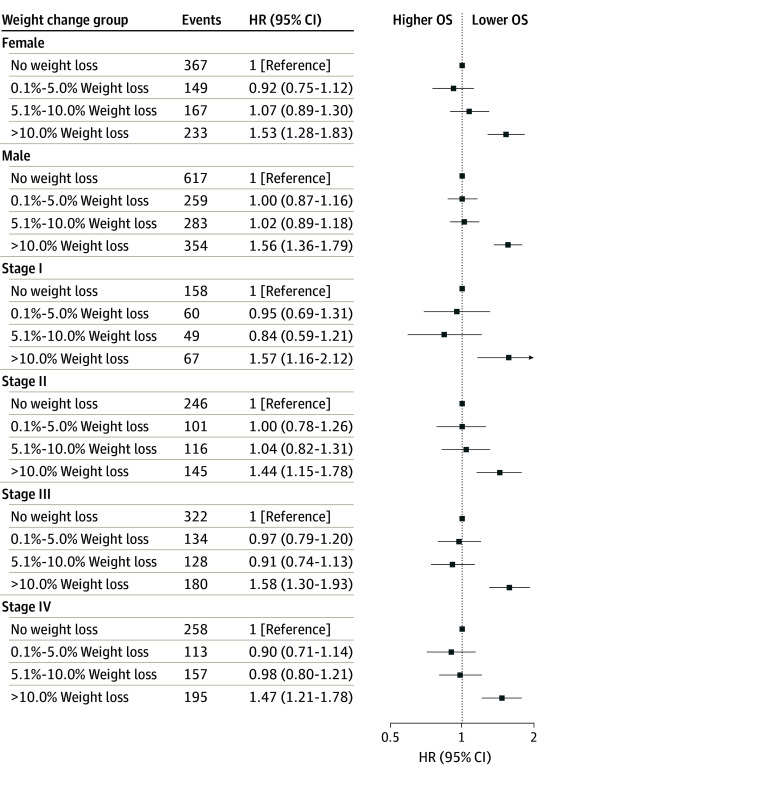
Descriptive Forest Plot of Weight Change Since 5 to 14 Years Before Diagnosis and Overall Survival (OS), Stratified by Sex and Stage Models adjusted for age, sex, time interval (years before diagnosis), tumor stage, tumor grade, tumor location, physical activity, smoking, nonsteroidal anti-inflammatory drugs and statin use, hyperlipidemia, history of cardiovascular disease (heart failure, myocardial infarction, angina pectoris, or stroke), diabetes, history of other cancer, and chemotherapy and radiotherapy. HR indicates hazard ratio

Sex- and CRC stage-specific estimates for OS are presented in Figure 2 and Figure 3 for BMI at diagnosis and prediagnostic weight change, respectively. A similar inverse association was observed for overweight (HR, 0.85; 95% CI, 0.73-0.99) but not obesity (HR, 0.92; 95% CI, 0.76-1.11) at diagnosis among female participants, and there was no association for overweight (HR, 0.92; 95% CI, 0.82-1.04) or obesity (HR, 0.92; 95% CI, 0.79-1.06) among male participants. Attenuation of the magnitude of these estimates after additional adjustment for prediagnostic weight loss was observed in both sexes. In stage-specific analysis, overweight at diagnosis was associated with a higher OS in patients with stage II (HR, 0.81; 95% CI, 0.67-0.98) and stage IV (HR, 0.81; 95% CI, 0.68-0.96) cancer. For prediagnostic weight change, HRs were similar for female and male patients, with significantly worse survival observed among patients who lost more than 10% of body weight before diagnosis (female: HR, 1.53; 95% CI, 1.28-1.83; male: HR, 1.56; 95% CI, 1.36-1.79). Comparable inverse associations for prediagnostic weight loss exceeding 10% were observed across CRC stages.

Association of BMI at diagnosis with CRC survival according to smoking status is shown in eTable 2 in Supplement 1. Significantly better OS was observed only for patients with overweight at diagnosis in never-smokers (HR, 0.83; 95% CI, 0.72-0.95). Worse survival was observed among former smokers (HR, 3.13; 95% CI, 1.88-5.22) and current smokers (HR, 1.94; 95% CI, 1.16-3.26) with underweight.

Sensitivity analyses yielded consistent findings. Restricting the analysis to patients with weight information from 5 to 7 years before diagnosis showed similar patterns but with greater uncertainty due to fewer cases (eTable 3 in Supplement 1). Excluding the first year of follow-up (landmark analysis) slightly attenuated the associations (eFigure 5 in Supplement 1), and excluding stage IV patients marginally changed estimates (eFigure 6 in Supplement 1). Compared with a trajectory of normal BMI to normal BMI, trajectories of normal BMI to underweight BMI (HR, 1.66; 95% CI, 1.23-2.24) and overweight BMI to normal BMI (HR, 1.35; 95% CI, 1.18-1.56) were associated with worse OS, whereas a trajectory of overweight BMI to overweight BMI showed no association (eTable 4 in Supplement 1).

## Discussion

In this large, population-based case-control study with long-term follow-up of patients with CRC, the apparent survival advantage associated with overweight and obesity at diagnosis largely disappeared after accounting for prediagnostic weight loss. In contrast, substantial prediagnostic weight loss (>10%) remained consistently associated with worse survival. These findings suggest that a substantial part of the obesity paradox reported in CRC survival studies may be explained by prediagnostic weight loss.

Excess adiposity has been identified as a risk factor for many different cancer types, including CRC.^4^ Proposed biological mechanisms include systemic chronic inflammation, abnormalities in the insulin and insulin-like growth factor signaling, and disbalance in the sex-hormone metabolism.^22^ Accordingly, excess adiposity might be expected to worsen survival in patients with CRC. However, evidence is conflicting, with some studies reporting better survival in patients with CRC with BMI in the overweight range, or even in the obesity range, relative to patients with normal weight. For example, 2 recent meta-analyses by Wen et al^23^ and Li et al^24^ found higher BMI to be associated with more favorable CRC outcomes. Conversely, meta-analyses by Jaspan et al^25^ and Petrelli et al^26^ reported obesity to be associated with increased risk of overall and CRC-specific mortality.

Higher survival in patients with CRC with overweight and obesity, as well as the heterogeneity of associations found in epidemiological studies, likely reflects a range of methodological factors^7,8^; these include reverse causation, varying timing of BMI measurement, study populations heterogeneity, selection bias, and confounding. Reverse causation appears to be a particularly compelling explanation for much of the observed paradox. The preclinical but detectable phase of CRC has been estimated to last 2 to 6 years,^18,19,20^ during which many patients experience weight loss.^9,10,11^ Consequently, BMI measured near the time of diagnosis may already be influenced by the disease, leading to downward shifts across BMI categories. Such shifts can create the appearance of a survival advantage among higher BMI categories. We have previously demonstrated that if prediagnostic weight loss is not considered in the analysis of the association of excess weight with CRC risk, it can lead to considerable attenuation or even reversal of the association.^12,27,28^ This seems an even more important consideration in CRC prognosis studies.

In the present analysis, while patients with overweight at diagnosis initially showed better OS compared with those with normal weight, this association was no longer evident in models mutually adjusted for prediagnostic weight change. In contrast, prediagnostic weight loss exceeding 10% was consistently associated with worse overall, CRC-specific, and recurrence-free survival. These findings are consistent with the direct analysis of BMI trajectories from 5 to 14 years before diagnosis: patients shifting from overweight to normal BMI had worse survival than those remaining in the normal category, whereas those remaining in the overweight category showed no association. The dose-response analyses further supported these findings, showing progressively worse survival with increasing weight loss. Although a smaller increase in risk was suggested at higher levels of weight gain, this was beyond the scope of the present study and warrants further research.

In the analysis by CRC stage, better OS was seen for patients with stage II and IV cancer who had overweight at diagnosis. Weight loss was more pronounced in patients with advanced-stage disease; however, the association of substantial weight loss with poorer survival was observed across all CRC stages, suggesting that weight loss captures prognostic information beyond tumor stage alone. Collider bias has also been suggested to contribute to the apparent obesity paradox.^29,30^ However, in our stratified analysis, similar associations were seen for overweight and obesity across smoking status. Given the limited number of events in some strata, these findings should be interpreted with caution.

Nevertheless, several previous studies and meta-analyses have interpreted the apparent survival advantage of overweight and obesity as evidence of a causal protective effect.^22^ Some have suggested that standard weight management recommendations should be reconsidered for patients with CRC with overweight or obesity.^31,32^ Such interpretations, however, are problematic in light of the strong possibility of reverse causation and related methodological issues.

Unfortunately, other measures of adiposity were not available in this study. Sarcopenia, defined as low muscle mass and function, is highly prevalent among CRC patients and is associated with worse survival, higher treatment-related toxicity, and increased recurrence risk.^33,34^ It is plausible that the observed lower mortality risk among patients with CRC in the overweight range at diagnosis may be in part attributable to higher volume of skeletal muscle, compared with individuals with lower BMI.^35,36^ Beyond body composition, lifestyle factors—particularly physical activity—represent promising intervention targets for improving CRC outcomes. Observational studies have found higher prediagnosis and postdiagnosis physical activity to be associated with better CRC survival outcomes,^37^ and the trial by Courneya et al^38^ demonstrated that structured exercise after chemotherapy leads to a longer disease-free survival and OS.

### Strengths and Limitations

Notable strengths of this study include its very large sample size, long and detailed follow-up of patients with minimal loss to follow-up (participant retention of 97%), and comprehensive adjustment for potential confounders. However, several important limitations of this analysis require consideration. First, body weight and height were self-reported, potentially introducing recall bias. Nonetheless, prior studies suggest that BMI derived from self-reports is generally underestimated.^39^ Second, prediagnostic weight change would have ideally been assessed uniformly since, for example, 5 years before diagnosis. However, due to the way body weight was recalled, weight was available at different intervals before diagnosis, which is why weight change since 5 to 14 years before diagnosis was used. Restricting the analysis to patients with weight information 5 to 7 years before diagnosis yielded similar results, albeit with wider confidence intervals due to fewer cases. Third, although the use of late-entry time helped reduce survivor bias, we cannot rule out its presence entirely. Fourth, BMI is not an optimal indicator of body composition because it cannot distinguish between fat mass and lean body mass, nor does it capture their distributions around the body.

## Conclusions

In this case-control study with prospective follow-up of patients with CRC, overweight and obesity at diagnosis were associated with better survival in unadjusted analyses, but these associations were attenuated after accounting for prediagnostic weight loss, implicating reverse causation as a major contributor to the obesity paradox in CRC. Apparent survival advantages associated with higher BMI at diagnosis should not be used as evidence to suggest that weight gain would be beneficial for patients with BMI in the normal range or to alter existing survivorship guidance. Instead, clinical assessment and prognostication should incorporate recent and long-term weight changes, and future research should prioritize detailed measures of body composition and randomized trials of targeted lifestyle interventions aimed at improving survival and quality of life among CRC survivors.

## Notes

Supplement 1. eTable 1. Available Information on Weight at Various Time Points According to Age at Diagnosis of Cases eMethods. Multiple Imputation eFigure 1. Distribution of BMI at Diagnosis and Weight Change Since 5-14 Years Before Diagnosis eFigure 2. Weight Change Since 5-14 Years by Interval (Years) eFigure 3. Weight Change Since 5-14 Years Before Diagnosis by CRC Stage eFigure 4. Restricted Cubic Splines—BMI at Diagnosis and Weight Change Since 5-14 Years Before Diagnosis and Survival eTable 2. BMI at Diagnosis and CRC Survival According to Smoking Status eTable 3. Analysis Restricted to Participants With Information Available for Weight Change Since 5-7 Years Before Diagnosis—Association of BMI at Diagnosis and Prediagnostic Weight Change With Survival eFigure 5. Association of BMI at Diagnosis With Survival—Exclusion of the First Year of Follow-Up eFigure 6. Association of BMI at Diagnosis With Survival—Exclusion of the Stage IV Patients eTable 4. BMI Trajectory From 5-14 Years Before Diagnosis and CRC Survival

Supplement 2. Data Sharing Statement
