# Supplementary material for: Body Mass Index at Diagnosis, Prediagnostic Weight Loss, and Survival in Colorectal Cancer
Source: JAMA Netw Open. 2026 Aug 14;9(8):e2629571. doi: 10.1001/jamanetworkopen.2026.29571 (PMC13476838; doi:10.1001/jamanetworkopen.2026.29571)
Supplement: Supplementary file 1 [file jamanetwopen-e2629571-s001.pdf]

Supplemental Online Content

Mandic M, Wankhede D, Safizadeh F, Hoffmeister M, Brenner H. Body mass index at diagnosis, prediagnostic weight loss, and survival in colorectal cancer. *JAMA Netw. Open*. 2026;9(8):e2629571. doi:10.1001/jamanetworkopen.2026.29571

eTable 1. Available Information on Weight at Various Time Points According to Age at Diagnosis of Cases

eMethods. Multiple Imputation

eFigure 1. Distribution of BMI at Diagnosis and Weight Change Since 5-14 Years Before Diagnosis

eFigure 2. Weight Change Since 5-14 Years by Interval (Years)

eFigure 3. Weight Change Since 5-14 Years Before Diagnosis by CRC Stage

eFigure 4. Restricted Cubic Splines—BMI at Diagnosis and Weight Change Since 5-14 Years Before Diagnosis and Survival

eTable 2. BMI at Diagnosis and CRC Survival According to Smoking Status

eTable 3. Analysis Restricted to Participants With Information Available for Weight Change Since 5-7 Years Before Diagnosis—Association of BMI at Diagnosis and Prediagnostic Weight Change With Survival

eFigure 5. Association of BMI at Diagnosis With Survival—Exclusion of the First Year of Follow-Up

eFigure 6. Association of BMI at Diagnosis With Survival—Exclusion of the Stage IV Patients

eTable 4. BMI Trajectory From 5-14 Years Before Diagnosis and CRC Survival

This supplemental material has been provided by the authors to give readers additional information about their work.

© 2026 Mandic M et al. *JAMA Network Open*.

eTable 1. Available information* on weight at various time points according to age at diagnosis of cases

**Ages at diagnosis or interview**	**Available weight information**															
	current	0-1 y ago	1-2 y ago	2-3 y ago	3-4 y ago	4-5 y ago	5-6 y ago	6-7 y ago	7-8 y ago	8-9 y ago	9-10 y ago	10-11 y ago	11-12 y ago	12-13 y ago	13-14 y ago	14-15 y ago
30, 40, 50, 60, 70, 80	X											X				
31, 41, 51, 61, 71, 81	X												X			
32, 42, 52, 62, 72, 82	X													X		
33, 43, 53, 63, 73, 83	X														X	
34, 44, 54, 64, 74, 84	X															X
35, 45, 55, 65, 75, 85	X						X									
36, 46, 56, 66, 76, 86	X							X								
37, 47, 57, 67, 77, 87	X								X							
38, 48, 58, 68, 78, 88	X									X						
39, 49, 59, 69, 79, 89	X										X					

Abbreviations: y = year(s).
X – Time of the weight information used for calculating weight change since 5-14 years before diagnosis.

For example, for a participant aged 55 years, weight at age 50 was used (weight change since 5 years before diagnosis), and for a participant aged 53 years, weight at age 40 was used (weight change since 13 years before diagnosis).

*A subset of patients recruited after April 2015 were also explicitely asked about their weights 5 and 10 years before diagnosis. For these patients, if weight at the last considered decennial age (marked with X in the table) was longer ago than weights that they reported for 5 or 10 years before diagnosis, we instead used weight that they reported for 5 years before diagnosis (or 10 years before diagnosis if weight at 5 years before diagnosis was missing). For example, if the participant was 59 years old and recruited in 2016, we calculated weight change by substracting current weight by weight at 5 years before diagnosis (at age 54).

© 2026 Mandic M et al. *JAMA Network Open*.

eMethods. Multiple imputation

We used multiple imputation by chained equations (20 iterations, 20 imputed datasets) for all variables included in the Cox models that had missing values. All variables used in the Cox models were included as predictors in the multiple imputation procedure. Continuous variables were imputed using predictive mean matching, binary variables using logistic regression, and categorical variables using multinomial or ordinal logistic regression as appropriate. Outcome variables (event indicators and follow-up times) were included as predictors in the imputation models, but were not imputed. This was done using R (mice) package.

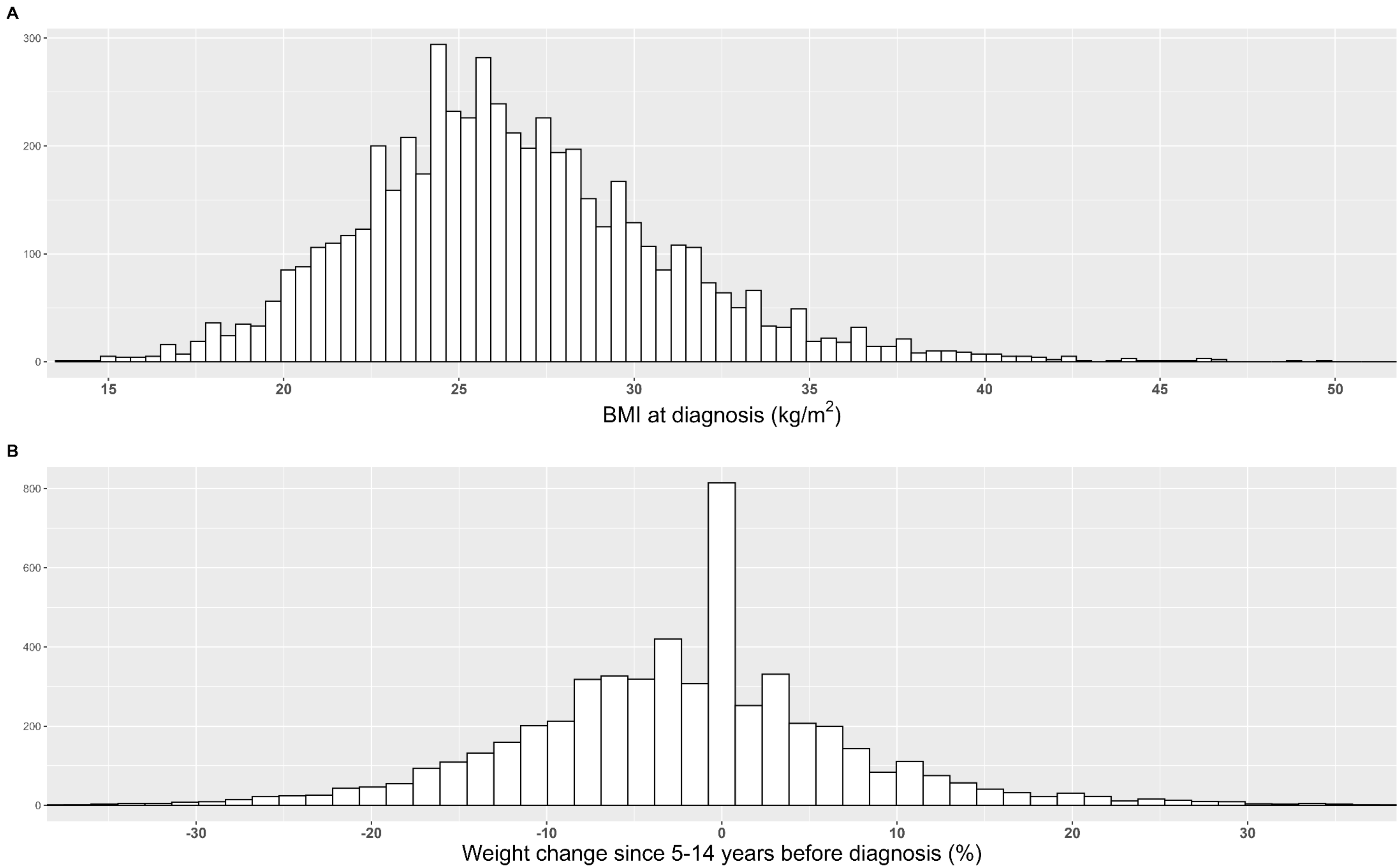

eFigure 1. Distribution of (A) BMI at diagnosis; (B) Weight change since 5-14 years before diagnosis (%)
Abbreviations: BMI = body mass index.

© 2026 Mandic M et al. *JAMA Network Open*.

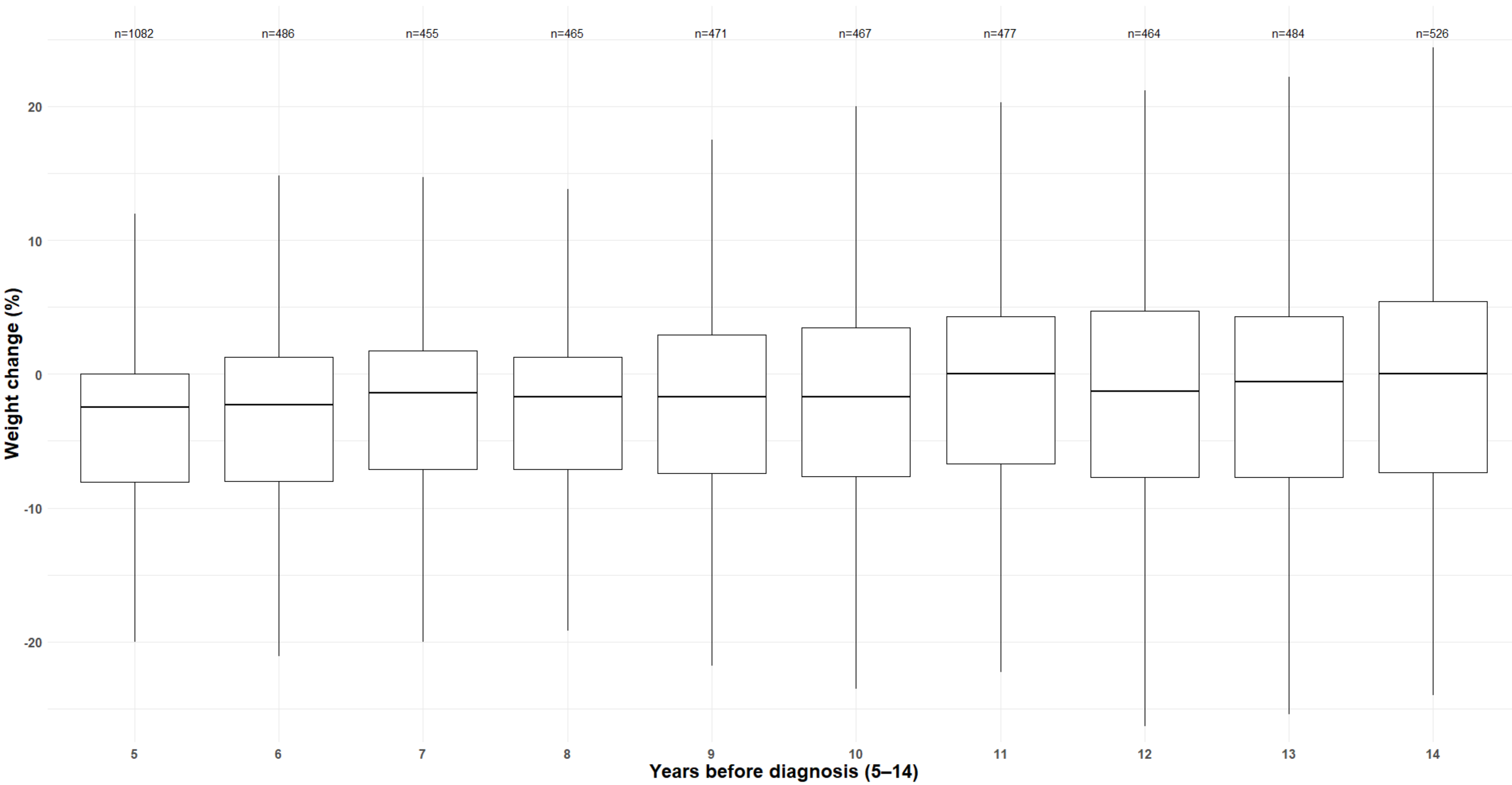

eFigure 2. Weight change since 5-14 years by interval (years)

© 2026 Mandic M et al. *JAMA Network Open*.

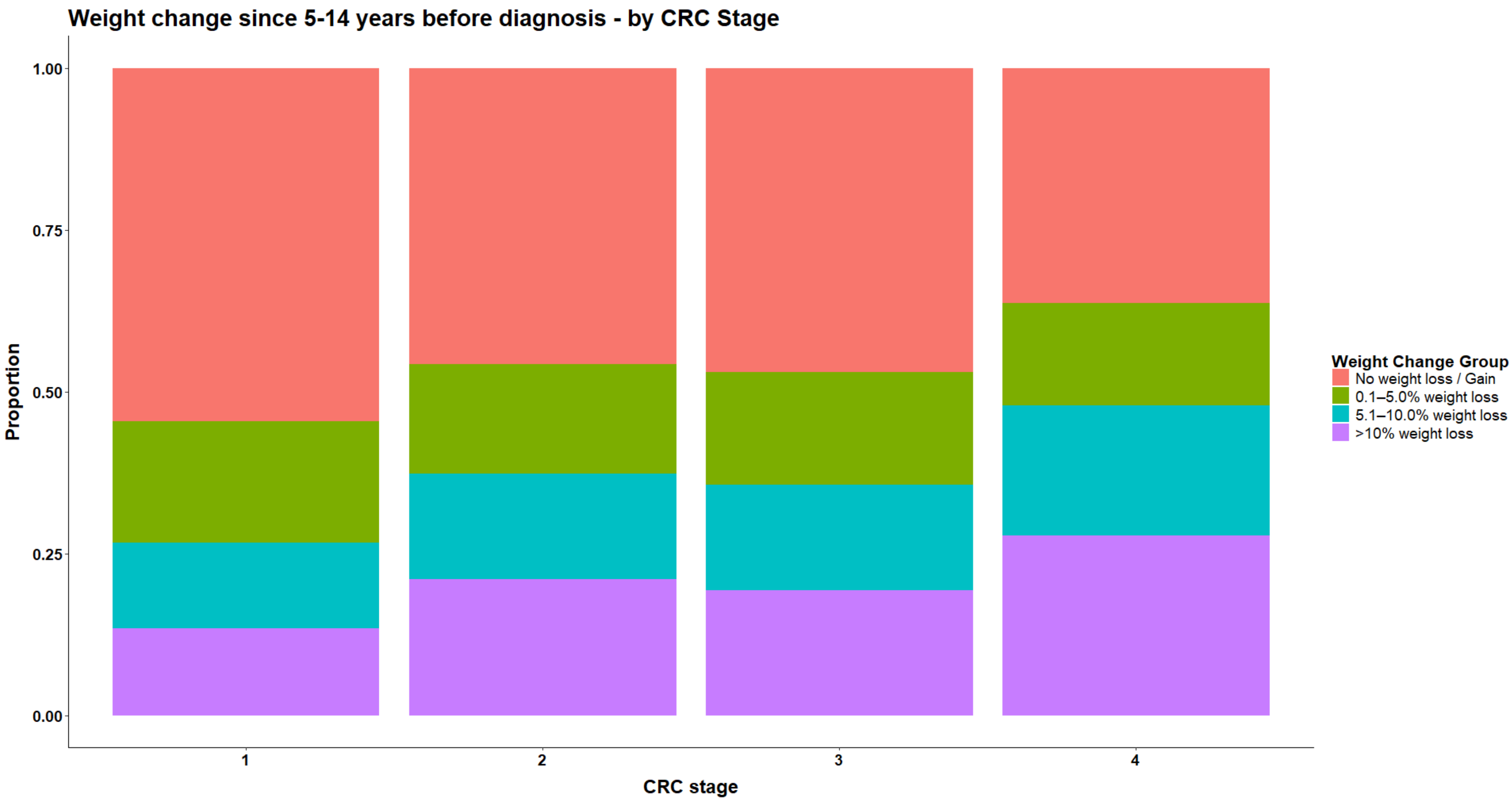

eFigure 3. Weight change since 5-14 years before diagnosis by CRC stage
Abbreviations: BMI = body mass index, CRC = colorectal cancer.

© 2026 Mandic M et al. *JAMA Network Open*.

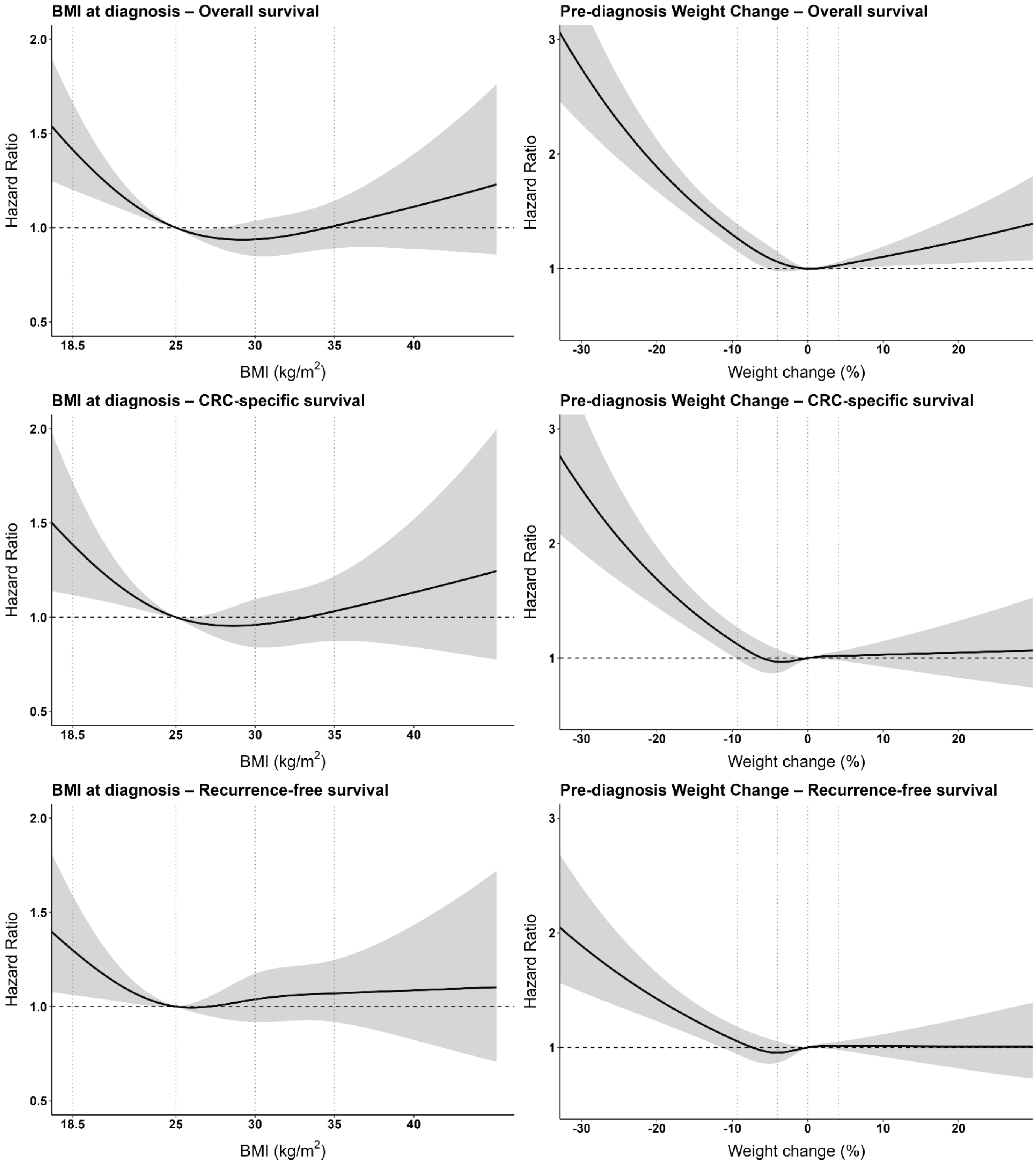

eFigure 4. Restricted cubic splines – BMI at diagnosis and weight change since 5-14 years before diagnosis and survival

Abbreviations: BMI = body mass index, CVD = cardiovascular disease, NSAIDs = nonsteroidal anti-inflammatory drugs.

For BMI - knots placed at 20, 25, 30, and 35 kg/m^2, and 25 kg/m^2 used as reference; For weight change: knots placed at 20th (-9.3%), 40th (-4.0%), 60th (0.0%) and 80th (4.1%) percentile, and no weight change (0%) used as reference. Models adjusted for age, sex, tumor stage, tumor grade, tumor location, physical activity, smoking, NSAIDs and statin use, hyperlipidemia, history of CVD (heart failure, myocardial infarction, angina pectoris, or stroke), diabetes, history of other cancer, and chemotherapy and radiotherapy. For weight change, additionally adjusted for the corresponding time interval (years before diagnosis).

© 2026 Mandic M et al. *JAMA Network Open*.

eTable 2. BMI at diagnosis and CRC survival according to smoking status

		Never-smokers				Previous smokers				Current smokers			
		Under-weight	Normal weight	Overweight	Obesity	Under-weight	Normal weight	Overweight	Obesity	Under-weight	Normal weight	Overweight	Obesity
	At risk	55	888	1038	479	23	725	982	479	36	373	312	131
Overall survival	Events, n	30	420	454	221	16	322	417	182	19	153	142	53
	aHR[1] (95% CI)	1.13 (0.78-1.64)	[Ref]	0.82 (0.72-0.94)	0.98 (0.83-1.16)	2.80 (1.69-4.62)	[Ref]	0.89 (0.77-1.03)	0.88 (0.73-1.06)	1.54 (0.94-2.53)	[Ref]	1.02 (0.81-1.29)	0.94 (0.69-1.29)
	aHR[2] (95% CI)	1.04 (0.72-1.51)	[Ref]	0.83 (0.72-0.95)	0.92 (0.77-1.09)	3.13 (1.88-5.22)	[Ref]	0.91 (0.78-1.05)	0.88 (0.73-1.07)	1.94 (1.16-3.26)	[Ref]	1.11 (0.88-1.41)	0.93 (0.67-1.29)
CRC-specific survival	Events, n	19	241	266	141	9	169	226	85	10	102	84	29
	aHR[1] (95% CI)	1.41 (0.88-2.26)	[Ref]	0.86 (0.72-1.02)	1.07 (0.87-1.32)	2.47 (1.26-4.90)	[Ref]	0.93 (0.76-1.14)	0.72 (0.55-0.94)	1.19 (0.62-2.30)	[Ref]	0.93 (0.70-1.25)	0.79 (0.52-1.20)
	aHR[2] (95% CI)	1.27 (0.79-2.04)	[Ref]	0.86 (0.72-1.02)	1.03 (0.83-1.28)	2.98 (1.50-5.92)	[Ref]	1.06 (0.86-1.30)	0.84 (0.64-1.11)	1.51 (0.76-3.00)	[Ref]	0.99 (0.73.1.34)	0.93 (0.60-1.44)
Recurrence free survival	Events, n	23	289	322	170	10	203	275	118	13	125	102	35
	aHR[1] (95% CI)	1.46 (0.95-2.24)	[Ref]	0.88 (0.75-1.04)	1.10 (0.90-1.34)	1.46 (0.95-2.24)	[Ref]	0.88 (0.75-1.04)	1.10 (0.90-1.34)	1.16 (0.64-2.11)	[Ref]	0.97 (0.74-1.28)	0.80 (0.54-1.17)
	aHR[2] (95% CI)	1.24 (0.81-1.91)	[Ref]	0.91 (0.77-1.08)	1.07 (0.89-1.34)	1.26 (0.82-1.94)	[Ref]	0.91 (0.77-1.08)	1.08 (0.88-1.32)	1.33 (0.72-2.49)	[Ref]	1.03 (0.77-1.36)	0.92 (0.62-1.38)

Abbreviations: aHR = adjusted hazard ratio, BMI = body mass index, CI = confidence interval, CRC = colorectal cancer, CVD = cardiovascular disease, NSAIDs = nonsteroidal anti-inflammatory drugs.

1. Adjusted for age and sex. Additional adjustment for age X log(time) to account for time-dependent effects.
2. Adjusted for age, sex, tumor stage, tumor grade, tumor location, physical activity, NSAIDs and statin use, hyperlipidemia, history of CVD (heart failure, myocardial infarction, angina pectoris, or stroke), diabetes, history of other cancer, and chemotherapy and radiotherapy.

© 2026 Mandic M et al. *JAMA Network Open*.

eTable 3. Analysis restricted to participants with information available for weight change since 5-7 years before diagnosis - Association of BMI at diagnosis and prediagnostic weight change with survival

	Categories	Patients, No.	Events, No.	Age- and sex-adj. HR[1] (95% CI)	Multivarible HR[2] (95% CI)	Mutually adjusted, multivariable HR[3] (95% CI)
**Overall survival**						
BMI at diagnosis (kg/m²)	<18.5	40	19	2.00 (1.24-3.20)	1.81 (1.12-2.91)	1.59 (0.98-2.59)
	18.5 to <25	722	276	[Reference]	[Reference]	[Reference]
	25 to <30	853	320	0.86 (0.73-1.01)	0.91 (0.77-1.07)	0.94 (0.80-1.12)
	≥30	439	162	0.95 (0.78-1.15)	0.94 (0.77-1.15)	1.01 (0.82-1.26)
Weight change since 5-14 years before diagnosis (%)	No weight loss	894	304	[Reference]	[Reference]	[Reference]
	0.1-5.0%	410	140	0.98 (0.81-1.20)	0.91 (0.74-1.11)	0.89 (0.72-1.09)
	5.1-10.0%	382	151	1.14 (0.93-1.38)	0.95 (0.78-1.16)	0.93 (0.76-1.14)
	>10.0%	368	182	1.77 (1.47-2.13)	1.46 (1.20-1.76)	1.40 (1.14-1.72)
**CRC-specific survival**						
BMI at diagnosis (kg/m²)	<18.5	40	11	1.48 (0.80-2.74)	1.35 (0.72-2.53)	1.21 (0.64-2.28)
	18.5 to <25	722	172	[Reference]	[Reference]	[Reference]
	25 to <30	853	185	0.87 (0.70-1.07)	0.92 (0.74-1.15)	0.96 (0.77-1.20)
	≥30	439	93	0.88 (0.68-1.13)	0.97 (0.74-1.26)	1.05 (0.79-1.38)
Weight change since 5-14 years before diagnosis (%)	No weight loss	894	180	[Reference]	[Reference]	[Reference]
	0.1-5.0%	410	75	0.90 (0.68-1.19)	0.79 (0.60-1.04)	0.79 (0.59-1.03)
	5.1-10.0%	382	92	1.25 (0.97-1.61)	0.88 (0.68-1.14)	0.87 (0.67-1.13)
	>10.0%	368	114	1.83 (1.44-2.32)	1.40 (1.10-1.79)	1.37 (1.06-1.78)
**Recurrence-free survival**						
BMI at diagnosis (kg/m²)	<18.5	40	15	1.81 (1.06-3.07)	1.78 (1.04-3.04)	1.63 (0.94-2.82)
	18.5 to <25	722	205	[Reference]	[Reference]	[Reference]
	25 to <30	853	227	0.89 (0.72-1.08)	0.96 (0.78-1.17)	0.98 (0.80-1.21)
	≥30	439	129	1.02 (0.82-1.28)	1.18 (0.93-1.49)	1.24 (0.96-1.58)
Weight change since 5-14 years before diagnosis (%)	No weight loss	894	232	[Reference]	[Reference]	[Reference]
	0.1-5.0%	410	104	0.98 (0.77-1.24)	0.89 (0.70-1.13)	0.90 (0.70-1.14)
	5.1-10.0%	382	110	1.14 (0.91-1.45)	0.84 (0.66-1.06)	0.85 (0.66-1.08)
	>10.0%	368	130	1.54 (1.23-1.93)	1.21 (0.96-1.53)	1.23 (0.97-1.57)

Abbreviations: BMI = body mass index, CI = confidence interval, CRC = colorectal cancer, CVD = cardiovascular disease, HR = hazard ratio, MET = metabolic equivalent of task, NSAIDs = nonsteroidal anti-inflammatory drugs.

1. Adjusted for age (continuous) and sex. Additional adjustment for age X log(time) to account for time-dependent effects.
2. Fully-adjusted model: adjusted for age, sex, cancer stage (I to IV), tumor location (colon, rectum), tumor grade (G1, G2, G3, G4, unknown), physical activity (continuous; average lifetime MET hours per week), smoking status (never, previous, current), NSAIDs use (yes, no; defined as current regular use of NSAIDs, including aspirin, ≥2 times per week), statin use (yes, no; current use at least 1 time per week), hyperlipidemia (yes, no), history of CVD (heart failure, myocardial infarction, angina pectoris, or stroke)(yes, no), diabetes (yes, no), history of other cancer (yes, no), and use of chemotherapy and radiotherapy (yes, no; administered before a relapse/metastasis). Time interval (years before diagnosis) included in models that include weight change since 5-14 years before diagnosis.
3. Fully adjusted and simultaneous adjustment for BMI at diagnosis and weight change since 5-14 years before diagnosis.

© 2026 Mandic M et al. *JAMA Network Open*.

BMI at diagnosis

	Events: Full follow-up	Events: 1st year excluded	HR (95% CI)
**Overall survival**			
Underweight	65	40	1.40 (1.09, 1.80)<br>1.18 (0.86, 1.63)
Normal weight	895	657	Reference
Overweight	1013	788	0.89 (0.81, 0.98)<br>0.92 (0.83, 1.02)
Obesity I	368	276	0.90 (0.79, 1.02)<br>0.94 (0.81, 1.09)
Obesity II-III	88	70	0.89 (0.71, 1.11)<br>0.97 (0.76, 1.25)
**CRC-specific survival**			
Underweight	38	20	1.48 (1.06, 2.06)<br>1.13 (0.72, 1.78)
Normal weight	512	357	Reference
Overweight	576	433	0.94 (0.83, 1.06)<br>1.02 (0.89, 1.18)
Obesity	202	152	0.92 (0.78, 1.09)<br>1.02 (0.84, 1.24)
Obesity II-III	53	43	1.00 (0.75, 1.33)<br>1.23 (0.89, 1.70)
**Recurrence-free survival**			
Underweight	47	22	1.45 (1.07, 1.98)<br>1.24 (0.81, 1.92)
Normal weight	618	359	Reference
Overweight	697	409	0.97 (0.87, 1.09)<br>0.98 (0.85, 1.13)
Obesity	259	137	1.04 (0.89, 1.21)<br>0.99 (0.81, 1.21)
Obesity II-III	64	42	0.99 (0.76, 1.29)<br>1.17 (0.85, 1.63)

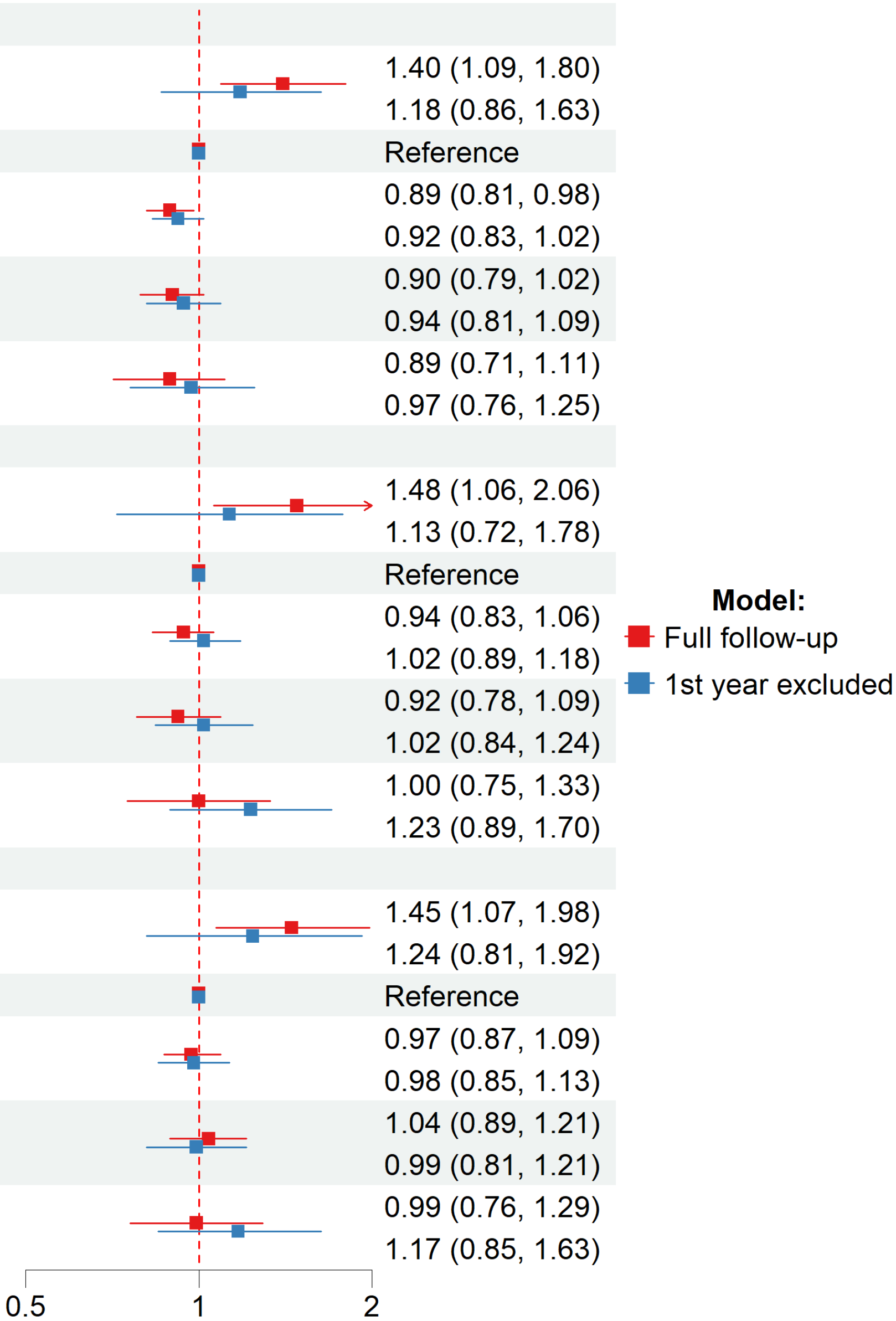

eFigure 5. Association of BMI at diagnosis with survival – <u>exclusion of the first year of follow-up</u>
Abbreviations: BMI = body mass index, CVD = cardiovascular disease, NSAIDs = nonsteroidal anti-inflammatory drugs.
Models adjusted for age, sex, tumor stage, tumor grade, tumor location, physical activity, smoking, NSAIDs and statin use, hyperlipidemia, history of CVD (heart failure, myocardial infarction, angina pectoris, or stroke), diabetes, history of other cancer, and chemotherapy and radiotherapy.

Red – Full follow-up included.
Blue – 1st year of follow-up excluded. Analysis restricted to participants who were alive and event-free at 365 days after diagnosis. Follow-up time was redefined as time since the 1-year landmark by subtracting 365 days from the original survival time.

© 2026 Mandic M et al. *JAMA Network Open*.

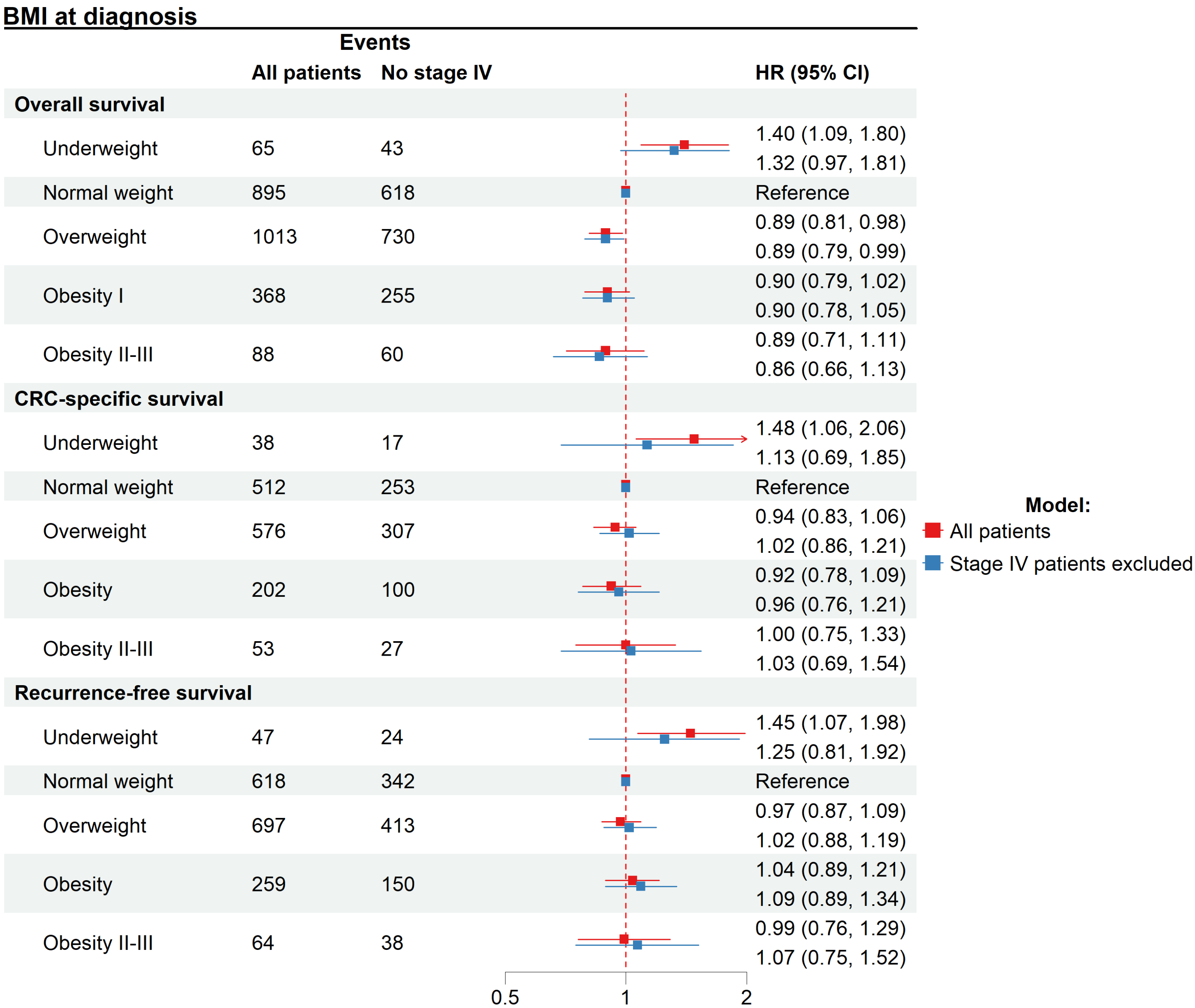

eFigure 6. Association of BMI at diagnosis with survival – exclusion of the stage IV patients

Abbreviations: BMI = body mass index, CVD = cardiovascular disease, NSAIDs = nonsteroidal anti-inflammatory drugs.
Models adjusted for age, sex, tumor stage, tumor grade, tumor location, physical activity, smoking, NSAIDs and statin use, hyperlipidemia, history of CVD (heart failure, myocardial infarction, angina pectoris, or stroke), diabetes, history of other cancer, and chemotherapy and radiotherapy.

Red – All participants (stages I-IV) included.
Blue – Restricted to stage I-III patients (stage IV excluded).

© 2026 Mandic M et al. *JAMA Network Open*.

eTable 4. BMI trajectory from 5-14 years before diagnosis and CRC survival

		Normal weight to Normal weight	Normal weight to Underweight	Overweight to Overweight	Overweight To Normal weight
	At risk	1299	82	1693	683
Overall survival	Events, n	513	47	733	384
	aHR[1] (95% CI)	[Ref]	1.66 (1.23-2.24)	0.95 (0.85-1.07)	1.42 (1.24-1.63)
	aHR[2] (95% CI)	[Ref]	1.42 (1.04-1.94)	0.96 (0.85-1.08)	1.35 (1.18-1.56)
CRC-specific survival	Events, n	307	27	407	202
	aHR[1] (95% CI)	[Ref]	1.64 (1.10-2.44)	0.97 (0.83-1.13)	1.40 (1.17-1.68)
	aHR[2] (95% CI)	[Ref]	1.68 (1.12-2.51)	0.99 (0.85-1.15)	1.36 (1.13-1.63)
Recurrence free survival	Events, n	378	31	502	238
	aHR[1] (95% CI)	[Ref]	1.52 (1.04-2.23)	1.02 (0.88-1.17)	1.38 (1.16-1.63)
	aHR[2] (95% CI)	[Ref]	1.52 (1.03-2.23)	1.05 (0.91-1.21)	1.30 (1.09-1.53)

Abbreviations: aHR = adjusted hazard ratio, BMI = body mass index, CI = confidence interval, CRC = colorectal cancer, CVD = cardiovascular disease, NSAIDs = nonsteroidal anti-inflammatory drugs.

Underweight: <18.5 kg/m^2; normal weight: 18.5 to 25 kg/m^2; overweight: 25 to 30 kg/m^2.

1. Adjusted for age and sex. Additional adjustment for age X log(time) to account for time-dependent effects.
2. Adjusted for age, sex, time interval (years before diagnosis), tumor stage, tumor grade, tumor location, physical activity, NSAIDs and statin use, hyperlipidemia, history of CVD (heart failure, myocardial infarction, angina pectoris, or stroke), diabetes, history of other cancer, and chemotherapy and radiotherapy.

© 2026 Mandic M et al. *JAMA Network Open*.
