# Supplementary material for: Body Mass Index at Diagnosis, Prediagnostic Weight Loss, and Survival in Colorectal Cancer
Source: JAMA Netw Open. 2026 Aug 14;9(8):e2629571. doi: 10.1001/jamanetworkopen.2026.29571 (PMC13476838; doi:10.1001/jamanetworkopen.2026.29571)
Supplement: Supplementary file 2 [file jamanetwopen-e2629571-s002.pdf]

Data Sharing Statement

Mandic. Body Mass Index at Diagnosis, Prediagnostic Weight Loss, and Survival in Colorectal Cancer. *JAMA Netw Open*. Published August 14, 2026.
doi:10.1001/jamanetworkopen.2026.29571

Data
Data available: No
